# Effect of Layer Charge Density on Hydration Properties of Montmorillonite: Molecular Dynamics Simulation and Experimental Study

**DOI:** 10.3390/ijms20163997

**Published:** 2019-08-16

**Authors:** Jun Qiu, Guoqing Li, Dongliang Liu, Shan Jiang, Guifang Wang, Ping Chen, Xiangnan Zhu, Geng Yao, Xiaodong Liu, Xianjun Lyu

**Affiliations:** 1 College of Chemical and Environmental Engineering, Shandong University of Science and Technology, Qingdao 266590, China; 2 School of Resources Environment and Materials, Guangxi University, Nanning 530004, China

**Keywords:** montmorillonite, layer charge density, hydration property, molecular dynamics simulation, experiment

## Abstract

Four kinds of Ca-montmorillonite with different layer charge density were used to study the effect of charge density on their hydration properties by molecular dynamics simulation and experiments. The research results of Z-density distribution of water molecules, H_w_ (hydrogen in water molecules), and Ca in the interlayer of montmorillonite show that the hydration properties of montmorillonite are closely related to its layer charge density. If the charge density is low, the water molecules in the interlayers are mainly concentrated on the sides of the central axis about –1.3 Å and 1.5 Å. As the charge density increases from 0.38_semi-cell_ to 0.69_semi-cell_, the water molecules are distributed −2.5 Å and 2.4 Å away from the siloxane surface (Si-O), the concentration of water molecules near the central axis decreases, and at the same time, Ca^2+^ appears to gradually shift from the vicinity of the central axis to the Si-O surface on both sides in the montmorillonite layer. The simulation results of the radial distribution function (RDF) of the Ca-H_w_, Ca-O_w_ (oxygen in water molecules), and Ca-O_t_ (the oxygen in the tetrahedron) show that the Ca^2+^ and O_w_ are more tightly packed together than that of H_w_; with the increase of the charge density, due to the fact that the negative charge sites on the Si-O surface increase, under the action of electrostatic attraction, some of the Ca^2+^ are pulled towards the Si-O surface, which is more obvious when the layer charge density of the montmorillonite is higher. The results of the RDF of the O_t_-H_w_ show that with the increase of charge density, the number of hydrogen bonds formed by O_t_ and H_w_ in the interlayers increase, and under the action of hydrogen bonding force, the water molecules near the central axis are pulled towards the two sides of Si-O surface. As a result, the arrangement of water molecules is more compact, and the structure is obvious. Correspondingly, the self-diffusion coefficient shows that the higher the layer charge density, the lower the self-diffusion coefficient of water molecules in interlayers is and the worse the hydration performance of montmorillonite. The experimental results of the experiments fit well with the above simulation results.

## 1. Introduction

Montmorillonite has a two-dimensional nano-scale layered structure [1]. Its TOT crystal structure unit is aluminum-oxygen octahedron in the middle and silica-oxygen tetrahedron in the upper and lower layers [2,3]. The interlayer of its TOT unit (so-called interlayer domain) usually contains a certain amount of water molecules and some exchangeable cations, so it has higher cation exchange capacity and higher water absorption and expansion capacity [4,5]. The hydration properties of montmorillonite have great influence on its application in various fields such as organic modification of montmorillonite, preparation of nanocomposite materials, drilling mud, pollution control, soil improvement, and others [6,7,8,9,10]; therefore, it is very important to study the hydration properties of montmorillonite.

Recently, with the development of science and technology, the methods of studying montmorillonite are not limited to experimental research. Computer molecular simulation can be used for theoretical research and experimental determination, which has been widely used in research of montmorillonite [11,12]. Compared with the traditional experimental research methods, the advantages of computer molecular simulation are obvious, which can not only simulate the molecular structure of the substance itself, but also simulate the dynamic change of the molecule when the substance reacts. It can visually describe the mechanism of reaction between substances at the molecular and atomic scales, and then verify the rationality of the experiment or predict the experimental results [13]. Skipper et al. first simulated the interlayer water structure of Na-montmorillonite and Mg-montmorillonite by using Monte Carlo (MC) and molecular dynamics methods (MD) in 1991, and proposed a complete MC simulation method for a clay–ion–water system in 1995, including the construction of the model, the selection of potential functions, and the processing of non-bonded interaction, which laid the foundation for the future molecular simulation study of montmorillonite [14,15]. Marry et al. studied the Na-montmorillonite and Cs-montmorillonite by MC and MD simulation methods, and compared their structural and kinetic characteristics with experimental data; their findings showed that the layer spacing calculated by molecular simulation, the diffusion coefficient of water molecules, and interlayer cations in a single layer hydrate were basically consistent with the experimentally measured data [16]. Therefore, MD simulation can be used as a new technology in the study of montmorillonite.

At present, molecular simulation technology has been successfully used to study the hydration characteristics of montmorillonite. Marry and Mignon studied the behavioral characteristics of different cations (Li^+^, Na^+^, and K^+^) in the hydration process of montmorillonite by methods of MC and MD simulation, and found that as the water content between the smectite layers increased, Li^+^ and Na^+^ were easily separated from the montmorillonite interlayer, while K^+^ moved to the surface of the siloxane tetrahedron and was bound to the surface [17,18,19]. Rahromostaqim compared the hydration and expansion properties of illite-montmorillonite (I-MMT) and Na-montmorillonite using molecular dynamics simulation, and found that at low CO_2_ concentrations in Na-MMT, which has its layers’ charge concentrated in its octahedral sheet, weak ion-surface interactions result in fully hydrated ions and, therefore, more extensive swelling than in I-MMT [20]. Most researchers believe that montmorillonite with stronger hydration ability layer cations (such as Na^+^, Li^+^) has higher expansion and hydrophilicity [21,22,23,24]. On the contrary, montmorillonite with weaker hydration cations (K^+^, Cs^+^) has lower swelling ability, poorer hydrophilicity, and its hydrophilicity is also affected by various factors (such as temperature, pressure, etc.) [5,25]. However, montmorillonite formed in different geological processes usually has different crystal chemical properties and layer charge characteristics, which will inevitably affect the hydration properties of montmorillonite, and therefore affect the physical and chemical performance of its derivatives [26]. As can be seen from the above, the previous studies in this field mainly focused on the influence of the interlayer cation types, temperature, and pressure conditions on hydration properties of montmorillonite [12,27,28]. Nevertheless, there are few studies about the effect of layer charge density on the hydration properties of montmorillonite.

In this work, four kinds of montmorillonite formed in different geological processes in China were used as experimental materials, and according to the chemical analysis results of purified montmorillonites, the position of all kinds of ions in montmorillonite crystals was determined, and the crystal-chemical formula was calculated. On this basis, the crystal structure models of four kinds of montmorillonite were established. The effect of layer charge density on its hydration characteristics was studied both by the methods of MD simulation and experiment.

## 2. Experiment

### 2.1. Materials

Four kinds of Ca-bentonite ores from Wei Fang, Lai Xi, and Inner Mongolia (two types) in China were purified according to the principle of Stokes settlement in water. The four purified montmorillonites were denoted as M1, M2, M3, and M4, respectively. The particle size of purified montmorillonite was controlled below 2 microns.

**Figure 1:**
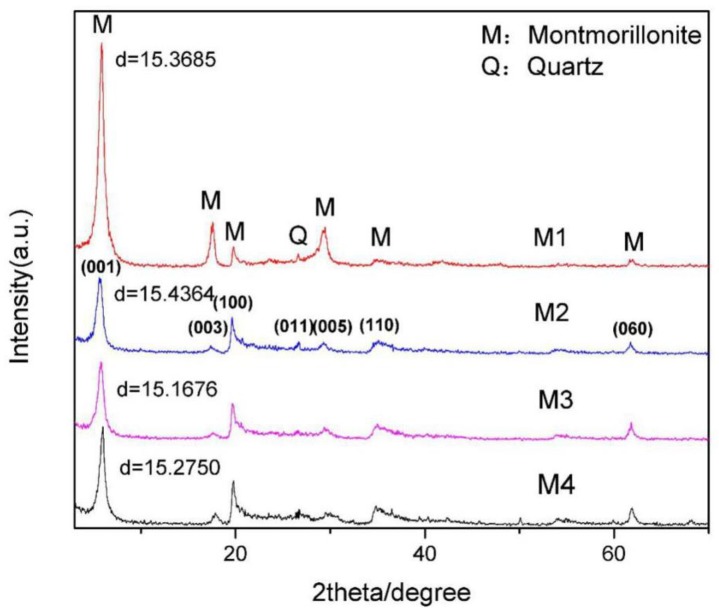
X-ray diffraction analysis of four kinds of purified montmorillonite.

The X-ray diffraction (XRD) spectrums of the four kinds of purified montmorillonite are shown in Figure 1, which shows that the four purified montmorillonites only contain few quartz impurities and can meet the requirements of crystal structure calculation. In addition, the chemical compositions of the four kinds of montmorillonite were analyzed by XRF. According to the chemical analysis results of montmorillonite (Table 1), the position of various ions in the lattice of montmorillonite and the corresponding crystal formulas were determined [29]. The calculation results are given in Table 2. The crystal chemical formulas of the four kinds of montmorillonite were as follows: (Ca_0.16_Mg_0.02_Na_0.01_K_0.01_)_0.38_(Si_3.98_Al_0.02_)_4_O_10_(Al_1.55_Mg_0.35_Fe_0.10_)_2_(OH)_2_, (Ca_0.25_K_0.01_)_0.51_(Si_3.97_Al_0.03_)_4_O_10_(A_1.52_Mg_0.43_Fe_0.05_)_2_(OH)_2_, (Ca_0.21_Mg_0.04_Na_0.05_K_0.02_)_0.57_(Si_3.94_Al_0.06_)_4_O_10_(Al_1.49_Mg_0.41_Fe_0.08_Mn_0.02_)_2_(OH)_2_, and (Ca_0.26_Mg_0.05_Na_0.04_K_0.03_)_0.69_(Si_3.94_Al_0.06_)_4_O_10_(Al_1.38_Mg_0.53_Fe_0.07_Mn_0.01_ Ti_0.02_)_1.99_(OH)_2_, respectively. Therefore, the semi-unit layer charge density of the four kinds of montmorillonite were 0.38, 0.51, 0.57, and 0.69, respectively.

### 2.2. Simulation Details

#### 2.2.1. Montmorillonite Models

In this work, the hydration properties of the four kinds of montmorillonite were simulated by Materials Studio7.0 simulation software (Accelrys, San Diego, CA, America) [23]. According to Table 1 and Table 2, the semi-unit crystal structure formula of montmorillonite was assumed to be M_x+y_(Al_2-x_Mg_x_)(Si_4-y_Al_y_)O_10_(OH)_2_ nH_2_O, where M is the exchangeable cations distributed between the montmorillonite layers(Ca^2+^), and x+y is the semi-unit layer charge density. The structure belongs to the monoclinic C2/m space group, the crystal layer is constant: a = 0.523 nm, b = 0.906 nm, and the c value is variable [19,30] when the structural unit layer is anhydrous, c = 0.960 nm; and if there are water molecules existing between the layers, the c value will vary with the amount of water molecules and the type of exchangeable cations between layers. Table 3 is the atomic coordinates of montmorillonite in the three-dimensional model. Based on these parameters, the montmorillonite model can be built. At present, most researchers use super-cells of 4a × 2b × 1c and 4a × 4b × 1c for simulation research [21,31]. Considering the research purpose and content of this work, 8a × 4b × 1c super large unit cell was established to simulate the hydration properties of montmorillonite, which can not only meet the needs of high-level layer charge montmorillonite modeling, but also ensure that the simulated layer charge is consistent with the actual experimental samples.

According to the types and contents of the elements of each montmorillonite in Table 2, the isomorphic substitution of montmorillonite was completed and crystal models with different charge were established. In order to reduce the effect of the types of substituted and interlaminar cations on hydration properties, the most abundant elements in tetrahedron and octahedron of montmorillonite were selected as the substitute cations, so Al replaced Si in tetrahedron and Mg replaced Al in octahedron, and the resulting negative charge was balanced by Ca^2+^ between layers. The homomorphic substitution was carried out according to the following conditions: The substitution position was randomly replaced, and the adjacent atoms could not be substituted at the same time in the octahedron or tetrahedral sheet [3]. Finally, four crystal models of montmorillonite with charge densities of 0.375, 0.500, 0.563, and 0.688 were established, which are basically consistent with the experimentally measured data and could be used for the simulation analysis in this paper.

The water molecules in the montmorillonite layers were added using the adsorption location module. Since the number of water molecules affects the interlayer spacing of montmorillonite, and when the interlayer cation of montmorillonite is Ca^2+^, the interlayer spacing is about 15 Å. So, we added 256 water molecules in layers of the four kinds of montmorillonite. After geometric optimization, the c value of the four kinds of montmorillonite were 15.4152 Å,15.4203 Å, 15.4126 Å, and 15.4134 Å, respectively, which was basically consistent with the measured d_(001)_ value by XRD (Figure 1) and could meet the basic requirements of simulation analysis.

#### 2.2.2. Simulation Parameter

In the process of crystal geometry optimization, it was assumed that the montmorillonite layer remained rigid, the unit cell parameters a, b, α, and γ remained unchanged, and both c and β were variable. This paper selected the smart minimizer algorithm, the parameters were set as follows: The Root Mean Square (RMS) force standard was 0.1 kcal/molÅ, the energy difference was 2 × 10^−5^ kcal/mol, and the RMS displacement standard was 1 × 10^−5^ Å. The RMS stress of the cells was 100 GPa. The long-range electrostatic action used the Ewald summation method; the short-range van der Waals used the atom-based summation method. The vacuum cutoff spacing was 12.5 Å [21], the spline width was 1 Å, the buffer width was 0.5 Å, and the number of iteration steps was 5000. 

Molecular dynamics simulation used Forcite module, the NPT and NVT system was selected, the temperature was 298.0 K, the step length was 0.5 fs, the total simulation time of NPT is 100 ps, and the total simulation time of NVT is 150 ps, the number of steps was 5 × 10^5^, the output was every 200 steps, and the force field was universal [32]. The Universal Force Field (UFF) can cover almost all the elements in the periodic table of elements, which can meet the requirements of this simulation study. During the simulation, all atoms in the interlayer domain were unconstrained, allowing atomic coordinates and lattice parameters to change freely, and the output data were used for result analysis.

### 2.3. Analytical Method

In the present work, X-ray diffraction (XRD) was used to analyze the mineral composition of montmorillonite. XRD was performed using a Bruker D8 Advance X-ray diffractometer with CuKα radiation, manufactured by RicoKu Co., Ltd. (Tokyo, Japan) The diffraction angle of the patterns was recorded from 3° to 70° with a scanning speed of 5°/min. The elemental quantitative analysis of the purified montmorillonite was carried out using the Axios advanced X-ray fluorescence spectrometer manufactured by PANalyticalB.V. (Almelo, Netherlands). The samples used for XRF analysis were made by casting.

Thermogravimetric analysis (TG) was used to study the thermal stability of montmorillonite and further study the effect of layer charge density on the interlayer water precipitation temperature of montmorillonite. Derivative thermogravimetric analysis (DTG) is a function of the rate of change of weight and temperature or time, which can accurately reflect the initial reaction temperature of samples. The combination of the two techniques can be used to study the thermal stability of montmorillonite and further study the effect of charge density on the water precipitation temperature between layers of montmorillonite. The TG analysis was carried out using a TG/SDTA851e thermogravimetric/differential thermal synchronizer, manufactured by Mettler-Toledo (Zurich, Switzerland), with a working gas of N_2_ and a heating rate of 5 °C/min. The starting temperature was 40 °C, and the end temperature was 300 °C; the sample was thoroughly dried at 100 °C for 12 h in advance.

## 3. Results and Discussion

### 3.1. The Overall Distribution Characteristics of Water Molecules in the Interlayer of Montmorillonite

The final configuration model can directly reflect the distribution of water molecules in the interlayer of montmorillonite. The Z-density distribution curves reflect the density distribution of each component in the direction of vertical montmorillonite surface in the interlayer. The combination can further study the distribution and relative position of water molecules in the interlayer of montmorillonite.

**Figure 2:**
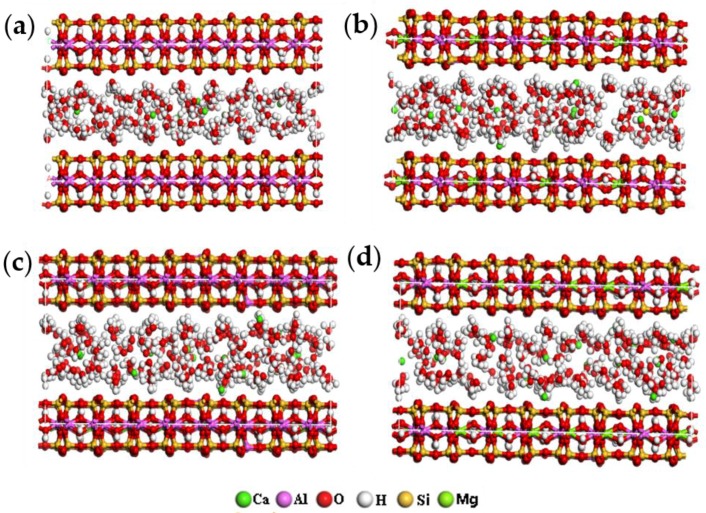
The hydration models of montmorillonite (ball-and-stick model) (**a**) 0.375; (**b**) 0.500; (**c**) 0.563; (**d**) 0.688.

According to the distribution characteristics of ions in the four types of montmorillonite in Table 1, combined with the detailed description in Section 2.2, the hydration models of the four kinds of montmorillonite with different charge density were finally optimized and shown in Figure 2.

As can be seen from Figure 2, with the increase of the charge density in the montmorillonite, the water molecules in the interlayer gradually evolved from a random disordered arrangement into H_w_ (H in the water molecules) turning to the siloxane surface of montmorillonite, while O_w_ (O in the water molecules) deviated from the siloxane surface. The above simulation results show that the charge density characteristics of montmorillonite has a significant influence on its hydration characteristics and interlayer ions distribution.

In this work, the molecular dynamics simulation software was used to study the influence mechanism of montmorillonite charge density on hydration performance by studying the Z-density distribution of water molecules, H_w_, and Ca^2+^ in the interlayer, the mean square displacement curve (MSD) and diffusion coefficient of water molecules, and the radial distribution function (RDF) of Ca-H_w_, Ca-O_w_, Ca-O_t_, O_t_-H_w_, and O_t_-Q_w_.

### 3.2. Z-density Distribution

#### 3.2.1. Z-density Distribution of Water Molecules and Hw in the Interlayer of Montmorillonite

**Figure 3:**
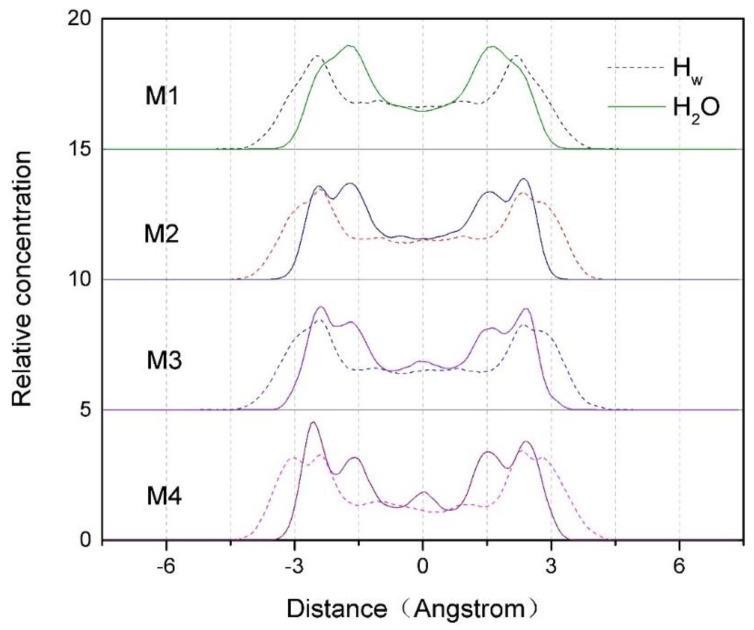
The Z-density profiles of water molecules and H_W_ within the interlayer spaces of montmorillonites with different charge density.

Figure 3 shows the Z-density profiles of water molecules and H_w_ in the interlayer spaces of montmorillonites with different charge density. As can be seen from Figure 3, the two distribution peaks of water molecules in the interlayer domain are located about –1.3 Å and 1.5 Å along the central axis, respectively, indicating that water molecules are mainly concentrated on both sides of the interlayer central axis of montmorillonite, while the two distribution peaks of H_w_ (H in water molecule) are located about –2.5 Å and 2.4 Å, which is about 0.9 Å closer to the Si-O surface than that of water molecules. It indicates that due to the hydrogen bonds force formed by H_w_ and O_t_ (O in the tetrahedron), the water molecules are deflected, so the H_w_ are closer to the Si-O surface than that of the water molecules.

In addition, according to Figure 3, we can also find that the layer charge density of montmorillonite has a significant effect on the distribution of water molecules in interlayer. When the charge density is low (such as M1), the water molecules are mainly distributed near the central axis, which is shown as two sharp peaks in the Figure 3. With the increase of charge density (such as M2 and M3), the main peaks on both sides of the central axis begin to split, and at the same time, the peaks get weaker. When the charge density increases to 0.688 (such as M4), two new peaks begin to form near about –2.5 Å and 2.4 Å, and so does the H_w_. The reason is that with the increase of the charge density, more and more hydrogen atoms in the water molecules turn to the Si-O surface, the number of hydrogen bonds formed by H_w_ and O_t_ (Table 4 and Figure 10) increases, and as a result, more and more water molecules are closely bound to the Si-O surface. However, as the montmorillonite charge density increases, a new small peak of water molecules appears near the axis; the reason is that the water molecules distributed in the middle of the interlayer domain are subjected to an equal hydrogen bonding force from both of the two Si-O surfaces; this phenomenon is more obvious when the layer charge density of montmorillonite is higher.

#### 3.2.2. Z-density Distribution of Ca^2+^ in the Interlayer of Montmorillonite

**Figure 4:**
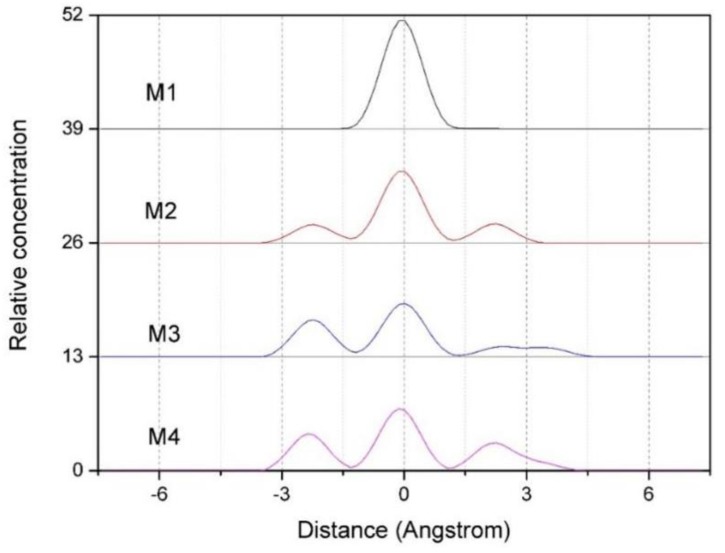
The Z-density profiles of Ca^2+^ in the interlayer of montmorillonite with different charge density.

Figure 4 shows the Z-density profiles of Ca^2+^ in the interlayer of montmorillonites with different charge density. As can be seen from Figure 4, the charge density has a significant influence on the distribution of Ca^2+^. With the increases in layer charge density, the Ca^2+^ appears to be gradually shift from the vicinity of the central axis to the Si-O surface on both sides in the montmorillonite layer, and this phenomenon is more obvious when the layer charge of montmorillonite is higher (such as M3, M4). The reason is that the Ca^2+^ between the layers of montmorillonite are subjected to electrostatic attraction of negative charges on the layers, causing the Ca^2+^ to diffuse to the Si-O surface. Since the montmorillonite with higher charge density has a large number of negative potential points, the Ca^2+^ in the interlayer are subjected to stronger electrostatic force; therefore, the diffusion of Ca^2+^ to the Si-O surface is more obvious. In general, the Z-density distribution of Ca^2+^ is similar to that of water molecules and H_w_ in water molecules.

### 3.3. Mean Square Displacement (MSD) Curves and the Diffusion Coefficient of Water Molecules

The MSD is the mean square of the change of particle position with respect to its initial position at different times, which can reflect the change of particle offset with time. The diffusion coefficient reflects the mobility of the particles, indicating the magnitude of the change of particle position with time, and its magnitude can be derived from the mean square displacement.

**Figure 5:**
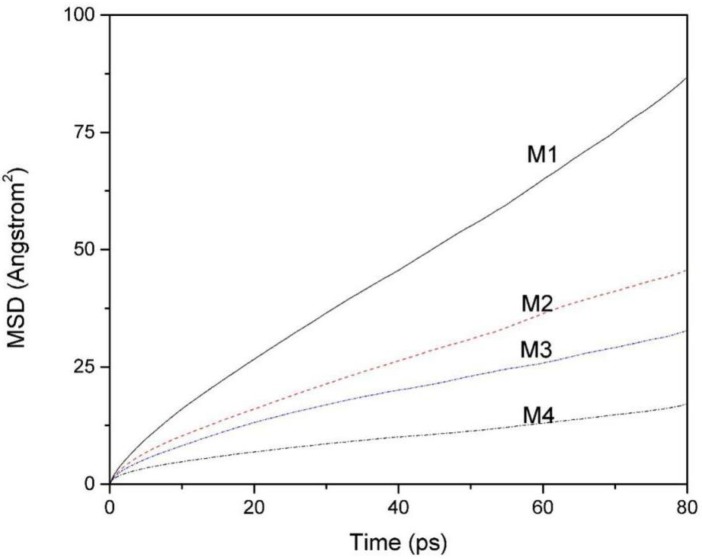
The mean square displacement (MSD) curves of water molecules of montmorillonites with different layer charge density.

Figure 5 is the MSD of water molecules of montmorillonites containing 256 water molecules with different layer charge density. The self-diffusion coefficient of water molecules in the montmorillonite interlayer can be derived from the MSD curves of water molecules. The magnitude of the diffusion coefficient is proportional to the slope of the MSD curves, i.e.: [33]

(1) $$D=\frac{1}{6N_{\alpha }}\underset{t\to \infty }{\lim}\frac{d}{dt}\sum_{i=1}^{N_{\alpha }}\left\{{\left[r_{i}\left(t\right)-r_{i}\left(0\right)\right]}^{2}\right\}$$

where ${\left[r_{i}\left(t\right)-r_{i}\left(0\right)\right]}^{2}$ is the mean square displacement of the molecule and *N_α_* is the total number of *α* particles. The equation shows that the self-diffusion coefficient *D* is 1/6 of the slope of the MSD curve.

It can be seen from Figure 5 that the MSD curves of the water molecules in the four kinds of montmorillonite are obviously different. Comparatively speaking, the mobility of water molecules in M1 is the highest and the mobility of water molecules in M4 is the lowest. According to formula (1), the calculated diffusion coefficient of water molecules **(**M1 to M4) is 1.67 × 10^−10^ m^2^/s, 0.87 × 10^−10^ m^2^/s, 0.58 × 10^−10^ m^2^/s, and 0.28 × 10^−10^ m^2^/s, respectively. Obviously, the diffusion coefficient of water molecules between layers decreases with the increase of charge density. The reason is that as the charge density of the layer increases, the number of hydrogen bonds formed between H_w_ and O_t_ increases, and more and more water molecules are bound to the Si-O surface, thus reducing its self-diffusion coefficient.

### 3.4. The Radial Distribution Function (RDF) of Ca-H_w_, Ca-O_w_, Ca-O_t_, O_t_-H_w_, and O_t_-Q_w_

The RDF reflects the probability density between two types of atoms and can reflect the aggregation characteristics of ions in the system. The RDF of β particles is evaluated as follows [33]:

(2) $$g_{\alpha \beta }\left(r\right)=n_{\beta }/4\pi \rho_{\beta }r^{2}dr$$

where g*_αβ_*(*r*) is the radial distribution of *β*, *n_β_* is the number of *β* with a radius of *r* → *r* + d*r*, *ρ_β_* is the number density of *β*, and *r* is the distance between *α* and *β*.

#### 3.4.1. The RDF of Ca-H_w_ and Ca-O_w_

**Figure 6:**
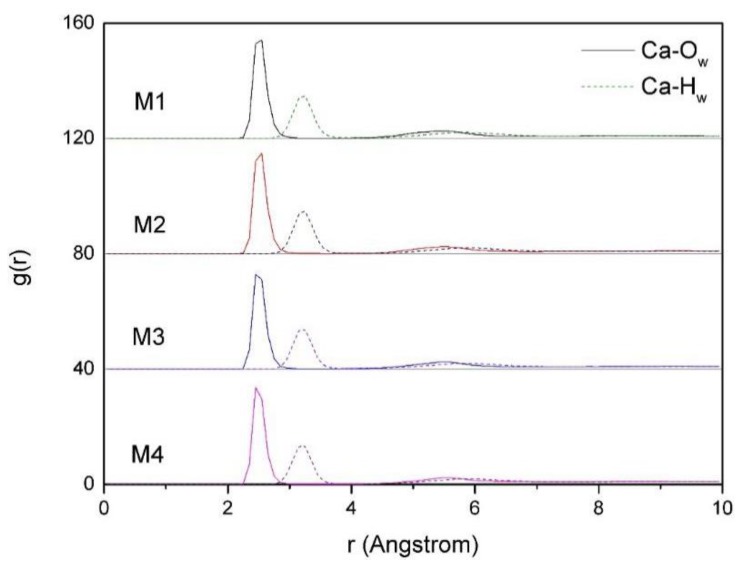
The radial distribution function (RDF) of Ca-O_w_ and Ca-H_w_ of montmorillonites with different layer charge density.

The RDF of Ca-H_w_ and Ca-O_w_ of montmorillonites with different charge density are shown in Figure 6. By studying the RDF of Ca-H_w_ and Ca-O_w_ in the interlayer of montmorillonites with different charge density, we can study the action of Ca^2+^ and water molecules between the layers of montmorillonite; therefore, the mechanism of the influence of Ca^2+^ on the distribution characteristics of water molecules can be further studied. It can be seen from Figure 6 that calcium ions are closely related to oxygen atoms in water molecules, the Ca^2+^ and the oxygen atoms in the water molecules are more tightly packed together than that of hydrogens so Ca^2+^ is more likely to have oxygen around than hydrogen in water molecules, and hydrated Ca^2+^ are formed near the Si-O surface. Comparatively speaking, it can be seen that there is little difference in the RDF of Ca-H_w_ and Ca-O_w_ characteristics with the increases of layer charge density.

#### 3.4.2. The RDF of Ca-O_t_

**Figure 7:**
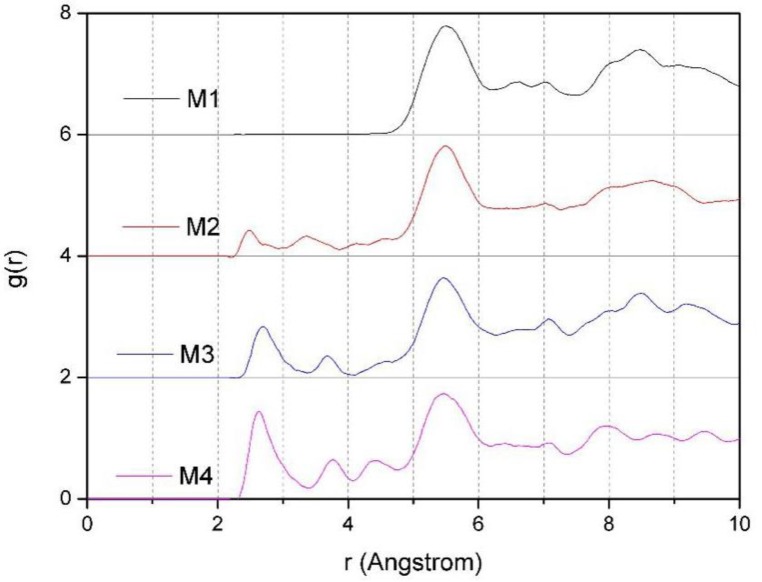
The RDF of Ca-O_t_ of montmorillonites with different layer charge density.

Figure 7 is the RDF of Ca-O_t_ of montmorillonites with different layer charge density. From Figure 7, we can find that with the increase in the charge density of montmorillonite, due to the fact that the negative charge sites on the Si-O surface increases, under the action of electrostatic attraction, some of the Ca^2+^ are pulled towards the Si-O surface; therefore, some new peaks appear around 2.6–4.5 Å, which is more obvious when the layer charge density of the montmorillonite is higher; this further indicates that calcium ions are close to the negative charge sites on the Si-O surface. The simulation results are consistent with Figure 2.

#### 3.4.3. The RDF of O_t_-H_w_ and O_t_-O_w_

**Figure 8:**
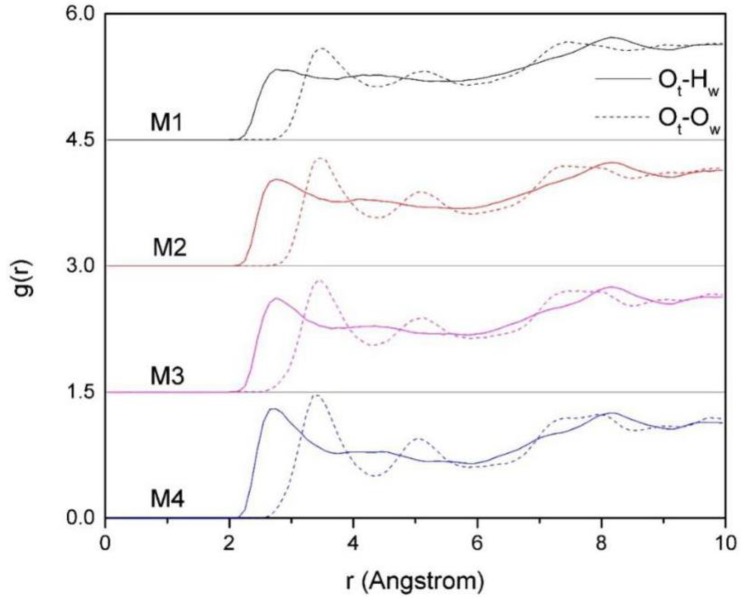
The RDF of O_t_-O_w_ and O_t_-H_w_ of montmorillonites with different layer charge density.

The RDF of O_t_-H_w_ and O_t_-O_w_ of montmorillonites with different charge density are shown in Figure 8. It can be seen that the maximum peak value of g (O_t_-H_w_) is approximately 2.5 Å, and the maximum peak value of g (O_t_-O_w_) is approximately 3.4 Å, which indicates that H_w_ is closer to the siloxane surface than O_w_ in water molecular. The reason is that the water molecules are polar molecules, the side of H_w_ is positively charged, and the side of O_w_ is negatively charged. On the other hand, the siloxane surface of montmorillonite is negatively charged, and due to the effect of the polar force, the H_w_ is close to the siloxane surface and forms hydrogen bonds with the O_t_. As a result, H_w_ is closer to the siloxane surface than O_w._ In addition, from Figure 8, we can also find that the effect of layer charge density of montmorillonite on the distribution of H_w_ and O_w_ in the layer is obvious. With the increases of charge density, the peaks of O_t_-H_w_ and O_t_-O_w_ get higher and steeper, which clearly indicates that the arrangement of H_w_, O_w_, and O_t_ is tight, and the arrangement of water molecules in the montmorillonite inter-layer is more compact.

**Figure 9:**
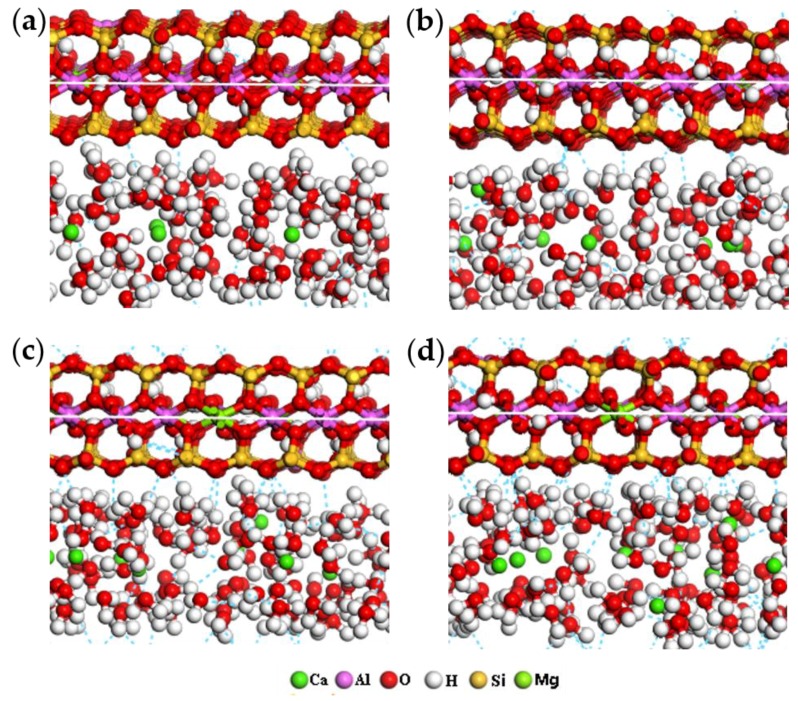
Schematic diagram of hydrogen bond of montmorillonites with different layer charge density (dashed line is hydrogen bond). (**a**) 0.375; (**b**) 0.500; (**c**) 0.563; (**d**) 0.688.

In order to further analyze the interaction between Si-O surface and water molecules in interlayer domain, we calculated the number of hydrogen bonds and the adsorption energy of Si-O surface to water molecules (Figure 9 and Table 4). It can be seen that the number of hydrogen bonds and the adsorption energy to water molecules also increase with the increase in the charge density of montmorillonite. It indicates that the water molecules in the montmorillonite with higher charge density are subjected to stronger polar force, and more and more H_w_ is diverted towards the Si-O surface, forming more hydrogen bonding with the O_t_. Due to the influence of the polar force and hydrogen bonding force, Si-O surface of montmorillonite with higher charge density has higher adsorption energy to water molecules, which further indicates that the higher the layer charge density of the montmorillonite, the more difficult it is to hydrate.

### 3.5. Experimental Study

The effect of layer charge density on water moleculars diffusivity of montmorillonite at the molecular and atomic levels has been studied by MD techniques. In order to macroscopically study the effect of charge density on hydration performance of montmorillonite, some experimental studies on the hydration performance of montmorillonite were conducted. TG and DTG were used to study the thermal stability and the difference in water precipitation temperature among montmorillonites. The swelling capacity and the gelling value were used to evaluate the swelling and dispersibility of montmorillonite.

#### 3.5.1. Thermogravimetric (TG) and Derivative Thermogravimetric Analysis (DTG)

The TG and DTG of montmorillonites with different layer charge densities are shown in Figure 9. The derivative thermogravimetric curve of montmorillonite shows two peaks. The first peak appears between 40–100 °C and this is the release temperature of the adsorbed water in the montmorillonite; the second peak appears between 100–200 °C and this is the precipitation temperature of the interlayer water in the montmorillonite. The reason for the different precipitation temperature of water molecules is that the interlayer water is hydrogen-bonded by Si-O surface, and the energy required for precipitation is higher than that of the adsorbed water. However, with the increase of temperature, the movement of interlayer water molecules is more severe, and the above-mentioned hydrogen bonding force formed by O_t_ and H_w_ is difficult to limit the movement of water molecules, thereby causing the interlayer water to precipitate.

**Figure 10:**
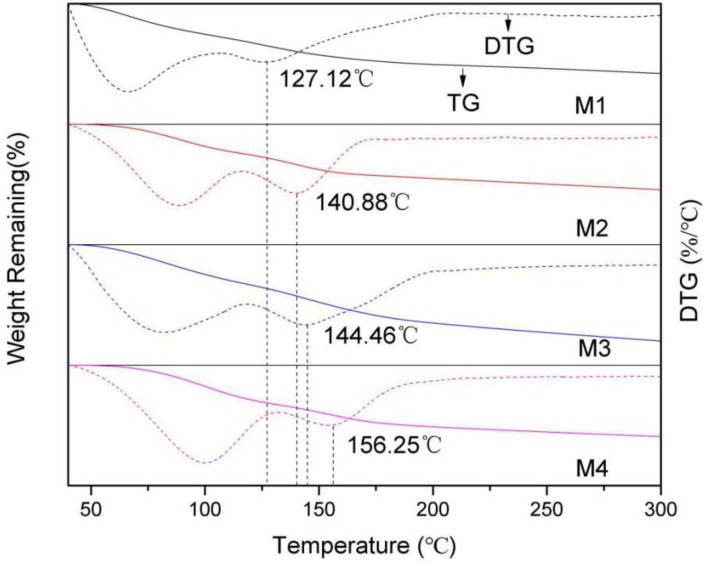
The TG and DTG curves of montmorillonites with different layer charge densities.

From Figure 10, we can also find that the maximum precipitation temperature of interlayer water molecules from M1 to M4 are 127.12 °C, 140.88 °C, 144.46 °C, and 156.25 °C, respectively, which indicates that as the charge density increases, the precipitation temperature of interlayer water of montmorillonite gradually increases. This is because the higher the layer charge density, the greater the number of hydrogen bonds formed by the H_w_ and the O_t_ (Table 3), the stronger the hydrogen bonding force on the interlayer water, the higher the energy required for the precipitation of water molecules, and the higher the temperature required for the precipitation of interlayer water.

#### 3.5.2. Swelling Capacity and Gelling Value of Montmorillonite

The hydration performance of montmorillonite includes swelling capacity and gelling value. The swelling capacity refers to the volume of montmorillonite after expansion in dilute hydrochloric acid solution. The gelling value is a comprehensive expression including the dispersibility, hydrophilicity, and swelling ability of the montmorillonite. Both of them can be used to characterize the hydration expansion properties of montmorillonite.

**Figure 11:**
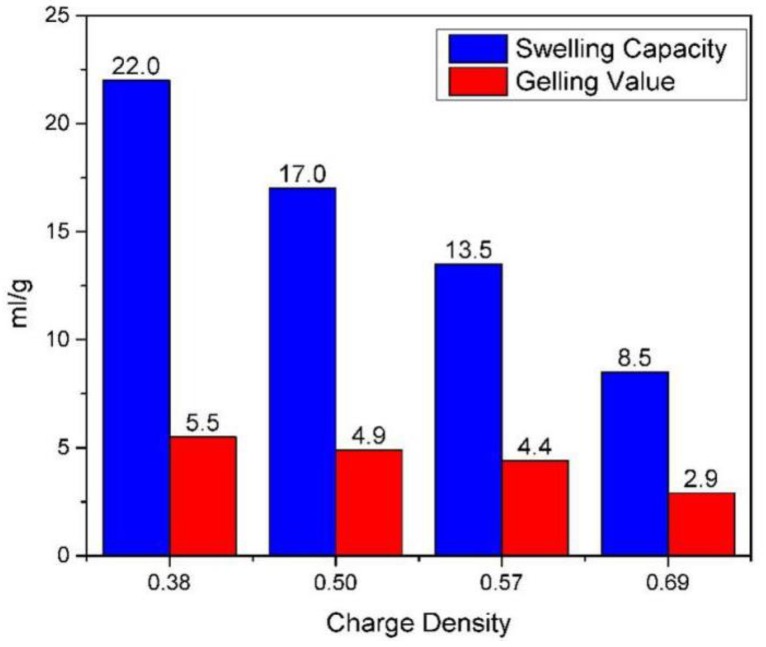
The swelling capacity and gelling value of montmorillonite with different layer charge density.

The swelling capacity and gelling values of the four kinds of montmorillonite were determined according to the literature [34,35] (Figure 11). As we can see from Figure 10, there are significant differences in the hydration properties of montmorillonites with different charge densities. The lower the layer charge density, the higher the swelling capacity and gelling values. The swelling capacity and gelling values of the montmorillonite with a charge density of 0.38 are 22 mL/g and 5.5 mL/g. The swelling capacity and gelling values of the montmorillonite with a charge density of 0.69 are 8.5 mL/g and 2.9ml/g, which indicates that as the charge density of montmorillonite increases, the hydration expansion and dispersion performance of montmorillonite decrease, which is consistent with the results of the previous molecular simulation.

## 4. Conclusions

In the present work, the hydration characteristics of four kinds of Ca-montmorillonite with different layer charge densities were studied by means of MD simulations and experiments. The research results indicate that the layer charge density of montmorillonite has a significant effect on hydration properties. If the charge density of montmorillonite is low (0.375), the water molecules are mainly concentrated on the sides of the central axis about –1.3 Å and 1.5 Å, and the self-diffusion coefficient of water molecules is only 1.67 × 10^−10^ m^2^/s. As the layer charge density increases (0.500, 0.563 to 0.688), the water molecules in the montmorillonite interlayer extends to –2.5 Å and 2.4 Å away from the central axis, and the self-diffusion coefficient of water molecules decreases to 0.87 × 10^−10^ m^2^/s, 0.58 × 10^−10^ m^2^/s, and0.28 × 10^−10^ m^2^/s, respectively. The concentration of water molecules in the central axis is reduced, the arrangement of water molecules is more compact, and the structure is obvious. With the increase of charge density, Ca^2+^ appears to gradually shift from the vicinity of the central axis to the Si-O surface on both sides in the montmorillonite layer, and the Ca^2+^ and O_w_ are more tightly packed together than that of H_w_. The reason is that with the increase of charge density of montmorillonite, the number of hydrogen bonds formed between the H_w_ and the O_t_ increases, more and more water molecules are bound to the Si-O surface, and under the action of electrostatic attraction, more and more Ca^2+^ are pulled towards the Si-O surface; this results in the higher the layer charge density of montmorillonite, the worse the hydration performance. The experimental results fit well with the simulation results.

## Figures and Tables

**Table 1:** Chemical composition of four kinds of purified montmorillonite.

Chemical Composition	Sample/%			
	M1	M2	M3	M4
SiO_2_	61.22	60.64	60.59	59.79
Al_2_O_3_	21.53	20.95	21.07	19.37
Fe_2_O_3_	1.03	0.57	0.85	0.75
MnO	0.00	0.00	0.18	0.09
TiO_2_	0.00	0.00	0.00	0.27
MgO	4.45	5.30	5.37	6.85
Na_2_O	0.10	0.00	0.46	0.36
CaO	1.61	2.44	2.17	2.57
K_2_O	0.06	0.08	0.13	0.27
Loss	9.87	10.26	9.33	9.82
Sum	99.87	100.24	100.15	100.14

**Table 2:** Calculation results of layer charge density of four kinds of montmorillonite.

Position	Cation	M1	M2	M3	M4
Tetrahedron	Si	3.98	3.97	3.94	3.94
	Al	0.02	0.03	0.06	0.06
	X_T_	–0.02	–0.03	–0.06	–0.06
Octahedron	Al	1.55	1.52	1.49	1.38
	Fe^2+^	0.1	0.05	0.08	0.07
	Mn^2+^	0	0	0.02	0.01
	Ti	0	0	0	0.02
	Mg	0.35	0.43	0.41	0.53
	X_O_	–0.36	–0.48	–0.51	–0.63
	Total	–0.38	–0.51	–0.57	–0.69
Interlayer Space	Na	0.01	0	0.05	0.04
	Ca	0.16	0.25	0.21	0.26
	K	0.01	0.01	0.02	0.03
	Mg	0.02	0	0.04	0.05
	X_L_	0.38	0.51	0.57	0.69

**Table 3:** Atomic coordinates of montmorillonite.

Atom	X	Y	Z
Al	0.000	3.020	15.500
Si	0.472	1.510	12.580
O	0.122	0.000	12.040
O	−0.686	2.615	12.240
O	0.772	5.510	14.200
O_(OH)_	0.808	4.530	14.250
H_(OH)_	−0.103	4.530	13.812

**Table 4:** The hydrogen bond number and adsorption energy of montmorillonites with different layer charge density.

Item	M1	M2	M3	M4
*n*	208	221	235	243
Adsorption Energy (kcal/mol)	−1478.047	−1670.016	−1736.206	−1857.187
